# Aphid-infested beans divert ant attendance from the rosy apple aphid in apple-bean intercropping

**DOI:** 10.1038/s41598-020-64973-7

**Published:** 2020-05-19

**Authors:** Joakim Pålsson, Mario Porcel, Mette Frimodt Hansen, Joachim Offenberg, Tiziana Nardin, Roberto Larcher, Marco Tasin

**Affiliations:** 1 Department of Plant Protection Biology, Swedish University of Agricultural Sciences, 230 53 Alnarp, Sweden; 2 Present Address: Corporación Colombiana de Investigación Agropecuaria (Agrosavia), C. I. La Libertad, Vía Puerto López km 17, Meta, Colombia; 3 Department of Bioscience, Plant and Insect Ecology, Aarhus University, 8600 Silkeborg, Denmark; 4 Technology Transfer Centre, Fondazione Edmund Mach (FEM), Via E. Mach 1, 38010 San Michele all’Adige, Italy; 5 Present Address: Department of Chemistry, University of Padova, Via Marzolo, 1 - 35121 Padova, Italy

**Keywords:** Ecology, Zoology

## Abstract

Ecological intensification of cropping systems aims at restoring multi-functionality while supporting current productivity levels. Intercropping is a form of ecological intensification involving ecological processes beneficial to farmers that do not take place in monocultures. Thus, it represents a practical approach to decrease the use of synthetic inputs such as insecticides in cultivated systems. Whereas insecticide reduction via intercropping-facilitated suppression of aphids is reported in literature, the majority of published studies focussed on herbaceous crops. Thus, the effect of intercropping on aphid populations of cultivated trees remains largely unaddressed. In this study we hypothesized that intercropping a specific companion plant within perennial crops would divert ant attendance from an aphid attacking the crop to another aphid feeding on the newly introduced plant, reducing aphid damage on the crop. We tested our hypothesis in the system of apple (*Malus domestica* Borkhausen), the rosy apple aphid (*Dysaphis plantaginea* Passerini) and the black garden ant (*Lasius niger* L.). Bean plants (*Vicia faba*) with the black bean aphid (*Aphis fabae* Scopoli) were intercropped within apple trees inoculated with *D. plantaginea*. We measured ant attendance, aphid development and survival as well as honeydew composition on both plant species through semi-field and field experiments. The majority of ants chose to attend *A. fabae* over *D. plantaginea* in the semi-field experiment with potted plants. In the orchard, a larger majority of scouts were scored on *A. fabae* over *D. plantaginea*. A higher number of *D. plantaginea* colonies remained active in the apple control, whilst they were almost eradicated by intercropping. Although chemical analyses of honeydew disclosed differences in the carbohydrate and amino acid profiles between aphid species, the difference in honeydew composition did not explain the preference for *A. fabae*. Ants did not discriminate between the two honeydew mimics both in laboratory and field bioassays. Our results showed the potential of intercropping apple trees with beans as a method to reduce ant attendance and thus colony survival. We propose that intercropping represents a bottom-up approach towards ecological intensification of perennial crops. Together with other ecosystem-based measures such as habitat management, intercropping should be considered when planning ecosystem redesign to increase biological control of pests.

## Introduction

Ecological intensification of agro-ecosystems is recognized as a fundamental change to restore multi-functionality while supporting high productivity levels^1^. It is predicted that the integration of ecosystem-based strategies with agro-ecological processes will allow for the development of productive and resilient farming systems^2^. Among various on-farm possibilities, such an objective can be achieved via improvement of the existing cropping systems, through both intraspecific and interspecific botanical diversification^3,4^. Benefits such as increased crop productivity, improved soil health and reduction of damage by biotic agents have been observed and measured in diversified crops^5–7^.

Intercropping is a form of botanical diversification that can trigger ecological processes that would otherwise not take place in a sole crop system, such as direct facilitation, niche complementarity in the use of plant resources, deterrence of pests and pathogens, and attraction of beneficial organisms^8^. For example, intercropping cereals with grain legumes promotes yield stability while it substantially reduces weed infestation and increases protein yield per hectare. In addition, an improved use of abiotic resources according to species complementarities for light interception and use of both soil mineral nitrogen and atmospheric nitrogen is documented^9,10^. Intercropping is also proposed as an approach to decrease pest damage through mechanisms like a reduced host finding, trap crop effects, the presence of deterrent plants, and the increased recruitment and survival of generalist natural enemies^11^. In this regard, there is very limited information on intercropping-mediated disruptions of mutualistic relationships of pests (i.e. ant-Hemiptera mutualism), resulting on a negative situation for the herbivores, and a potential pest damage reduction.

The use of companion plants in intercropped systems to reduce pest infestation was recently reviewed by Ben-Issa *et al*.^12^ who pointed out that in order to achieve long-term results, companion plant strategies need to be combined with other approaches against sucking pests such as aphids. However, intercropping alone reduced also aphid infestation in cotton, wheat and other crops^13–15^.

Although intercropping-mediated suppression of aphids is reported in literature, the majority of these studies focussed on herbaceous crops. Thus, the effect of intercropping on aphid populations of cultivated fruit trees remains largely unaddressed. A higher degree of trophic complexity, due to both longer longevity of the main crop and co-occurrence of non-crop species in the alleys in comparison with annual crops, may explain the lower number of studies in perennial systems. Among interactions, interspecific relations between ants and tree infesting aphids are often of mutualistic nature^16^. Plasticity in behaviour is found in some ant species, with plant chemical defence indirectly mediating aphid performance via interactions with tending ants^17^. Whereas ants harvest honeydew from the aphid colony as a source of carbohydrates and amino acids, aphids are protected by ants from consumption by natural enemies. The suppression of aphid populations via intercropping should thus not only consider effects such as dilution and spatial diversification of the crop, trap crop or attraction of antagonists, but also other effects such as ant diversion from the target aphid by dedicated intercropped plants. Mechanisms behind diversion include preferential attraction of ants towards the honeydew of an aphid pest, which attacks the specific intercropped plant. Such a behavioural preference may be driven by chemistry, with compounds such as trisaccharide sugars or amino acids triggering a higher attraction to the honeydew of the intercropped plant in comparison with that of the main crop. However, factors such as the size of aphid colonies and the related amount of produced honeydew as well as plant architecture and the distance of the aphid colony from the ant nest may also play a role.

In perennial cultivated systems such as apple orchards, several species of aphids inflict serious injury, requiring specific interventions with systemic aphicides like neonicotinoids. Although pesticides could be replaced by native natural enemies to keep aphid population at bay^18^, attendance by ants has been shown to push efficient predators away from aphid colonies^19^.

In this study, we hypothesized that introducing a specific plant within a tree crop would divert ant attendance from an aphid attacking the tree main crop to another aphid feeding on the newly added plant, contributing to an aphid reduction in the crop. We tested our hypothesis in the perennial system of apple (*Malus domestica* Borkhusen), where the rosy apple aphid *Dysaphis plantaginea* Passerini is a major pest^20^. Bean plants (*Vicia faba* L.) were inoculated with the black bean aphid (*Aphis fabae* Scopoli) and intercropped within apple trees inoculated with *D. plantaginea*. Bean and black bean aphid were chosen because of their phenological co-occurrence in the field with rosy apple aphid in this region. Apple as a sole crop was used as untreated control.

## Methods

In order to assess the effect of intercropping on ant-aphid interactions we carried out a series of sequential complementary experiments. Firstly, we tested in a semi-field setup whether *A. fabae* on bean could outcompete *D. plantaginea* on apple for ant attendance (Choice experiment in semi-field condition). Following this experiment, a field trial was carried out to corroborate our semi-field observations in the field and determine if there was an increased biological control of *D. plantaginea* (Field experiment). Additionally, we tested if the differences in attraction observed in the semi- and field experiments could be explained through differences in honeydew chemical composition, collecting and analysing the honeydew from both aphids (Chemical analyses), and testing the choice of ants to honeydew mimics (Laboratory and field choice test with honeydew mimic).

### Choice experiment in semi-field condition

Single faba beans cv. Gloria were sown into 2 l plastic containers filled with a vegetable substrate (Hasselfors Garden organic-certified, Sweden). *A. fabae* colonies originated from their winter host *Euonymus europaeus* L.. The migration of *A. fabae* from its winter host coincides with apple bloom and approximately the time when *D. plantaginea* colonies begin to develop in Sweden. Branches from three different plants of *E. europaeus* with *A. fabae* individuals were brought into contact with five-week-old *V. faba* plants to obtain new colonies. *A. fabae* colonized the bean plants after two weeks. Following the removal of any predator from the bean plants, a single *A. fabae* female was transferred to the top of newly potted beans to create an aphid colony per plant on a total of 30 plants. The main stem of each plant including the newly established colony was covered with a perforated plastic bag as a protection measure against predators. Plants were placed in a net-house to prevent aphid predation and used in behavioural experiments seven days after infestation.

Forty two-year-old potted apple trees (cv. Aroma) were purchased from Stångby Plantskola (AB, Lund, Sweden) in the spring of 2014 and maintained in 15 l pots. Trees were fertilized and pruned once a year. Pests were controlled by removing the insects manually after visual inspection. No disease control was required. Whereas a first batch of 20 trees was kept in a net-house (to avoid ant nesting), a second batch of 50 trees was placed in different apple orchards within the Skåne region to allow *L. niger* to nest inside the pot at the base of the trees. Worker ants were collected from each nest and observed under stereomicroscope to ascertain that the nest belonged to the species *L. niger*.

Only the rooting system of trees exposed in the orchards was used in further experiments. After cutting off the tree canopy at 10 cm from the ground, the presence or absence of an active nest with a queen and eggs was verified by inspecting the soil of each pot in the spring 2015. The root system with the ant nest was then removed in a block from the pot and transferred to an ant-secured starving-cardboard box (57.6 × 34.6 × 40.7 cm). Ants were kept without food and water during 14 hours before each run of the experiment. The potted trees that were not exposed in orchards, and therefore had no ant nests, were treated with Loxiran Myr (natural Pyrethrin extract, Neudorf, Emmerthal, Germany) in order to avoid the presence of any undetected ants.

**Figure 1:**
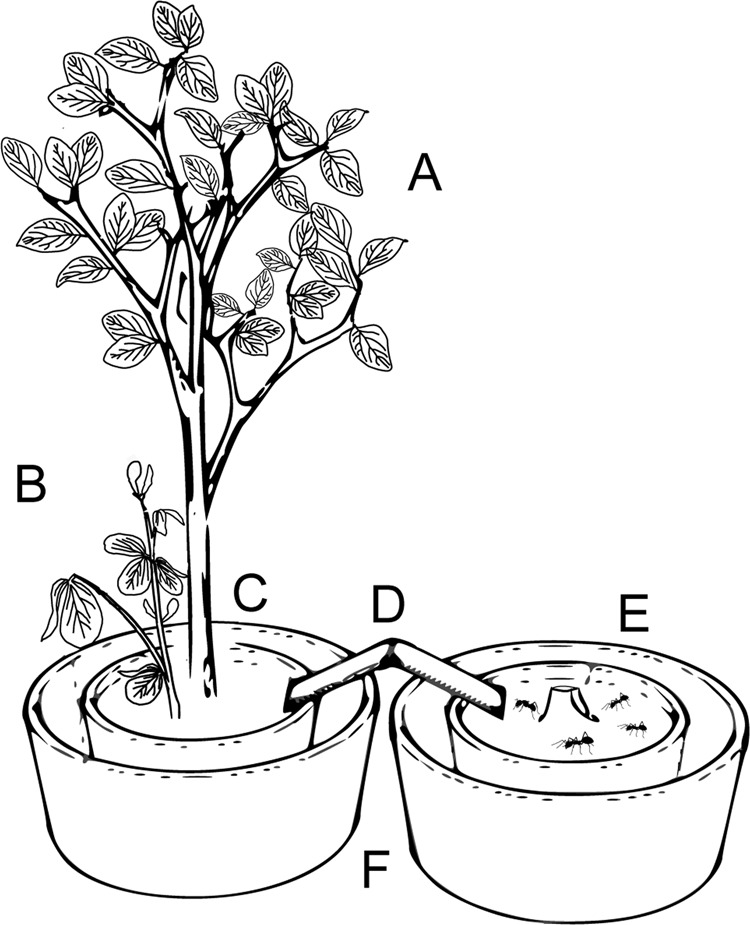
Schematic representation of the greenhouse choice assay. (**A**) apple tree with a *D. plantaginea* colony, (**B**) bean plant with a *A. fabae* colony, (**C**) choice arena (**D**) wooden bridge connecting the pot containing the ant nest to the choice arena, (**E**) pot containing the *L. niger* nest, and (**F**) masonry buckets filled with water.

A single *D. plantaginea* apterous virginoparae adult was collected from a field colony (Alnarp, SLU) and inoculated on a shoot of a potted tree using a clipcage (see Porcel *et al*.^18^ for details). Bean and apple plants were inoculated with the respective aphid on the same day. At day seven after inoculation, a *V. faba* plant with an established *A. fabae* colony was removed from its protective bag and planted into the same pot as the apple tree, avoiding a direct contact between the canopy of the apple tree and the bean. The clipcage around *D. plantaginea* was removed after planting the bean. Aphid colonies with similar size were selected on the paired plants. In order to prevent uncontrolled ant intrusion from outside, the pot with the two plants was placed inside a masonry bucket with water. At 8:00, ant nests were removed from the starving box and placed inside pots. This pot was then moved into a masonry bucket with water to avoid unwanted ant dispersal in the greenhouse. At 10:00 a.m., the two pots were connected with a wooden bridge, allowing the ants to freely access the pot hosting the two plants. The bridge was situated equidistantly between the apple tree and the bean plant (Fig. 1).

The number of *D. plantaginea* aphids on apple, *A. fabae* on bean, and ants tending colonies were counted six times in 2 h intervals from 10.00 a.m. to 8.00 p.m. Ants observed inside and around the aphid colonies were considered tending ants. Ten replicates were run simultaneously each day for three consecutive days using ten different *L. niger* nests. Each apple and bean plant was used once and each ant nest was used three times on different days. After each run of the experiment, nests were placed back into the starving boxes until the following day. Ants that had established aphid attendance were not returned to the nests. As a total of seven *D. plantaginea* colonies failed to establish in the first three days, a fourth run was carried out with the same seven sets of plants for a total of 30 replicates.

The number of ants attending *D. plantaginea* and *A. fabae* was analysed with a generalized mixed model (GLMM) with a log link and a Poisson error distribution using the R package “lmer4”. Aphid species and the interaction between Aphid species and Time were included as fixed factors. The interaction was dropped from the final model based on AIC goodness of fit. Ant nest and Day of the experiment were added as random effects. The replicate was included as a random effect to account for the dependency structure derived from repeated counts on the same colony over time. The probability of ant presence in *D. plantaginea* colonies was analysed with a binomial GLMM with the same fixed and random effects. The significance of the fixed factors was tested with Wald tests for both models. Final models were checked for overdispersion and the assumptions were verified by representing graphically the residuals against the fitted values, the fixed factors and the random effects. All statistical analyses described herein were carried out with R v. 3.5.2.

### Field experiment

**Figure 2:**
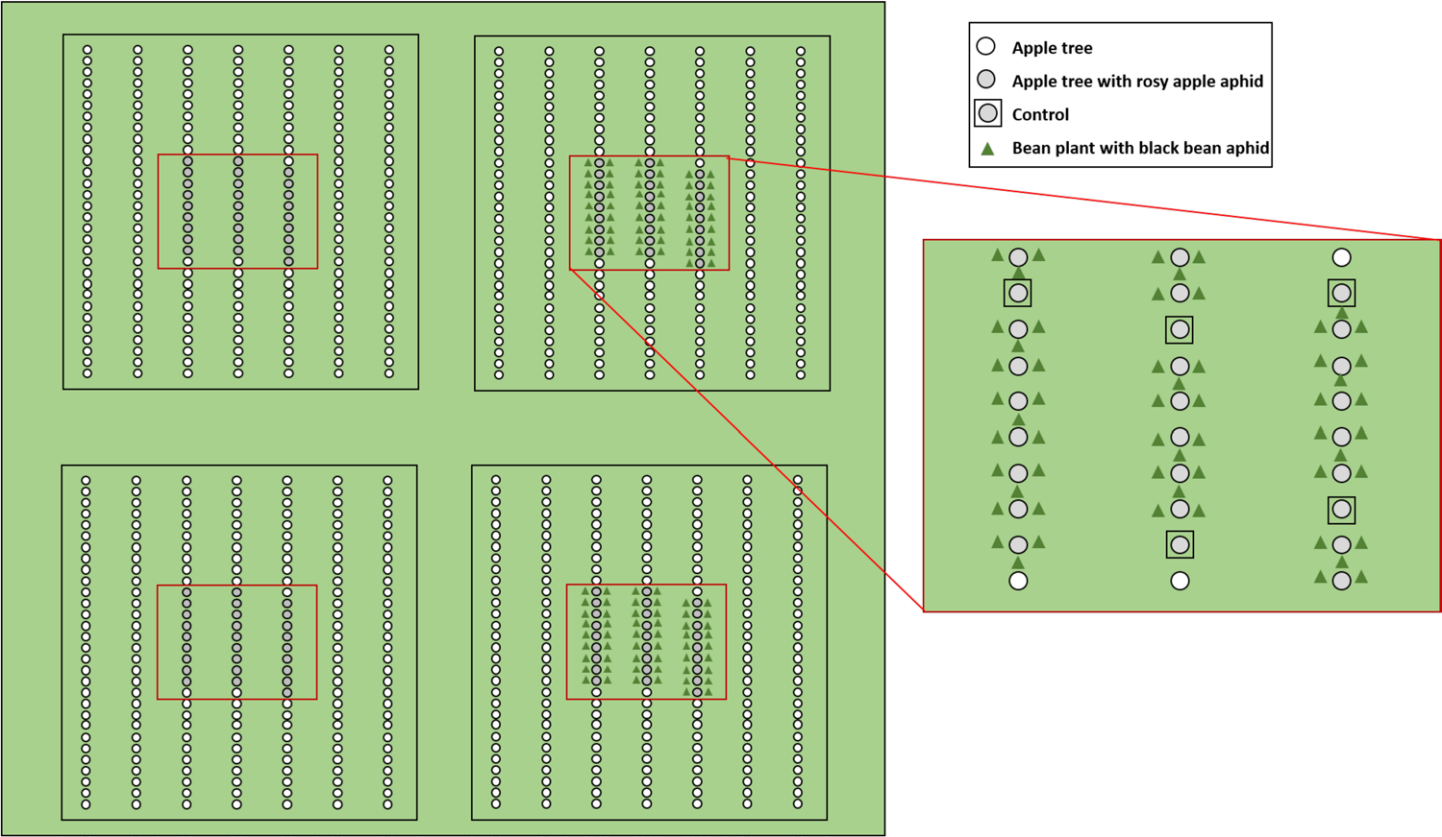
Schematic representation of the intercropping experiment in the orchard with apple trees and beans.

Four square plots (30 × 30 m) were established in an apple orchard in the Skåne region (Sweden). Plots included seven apple rows and were spaced a minimum of 25 m apart. The 12^th^ of June 2015, twenty-seven trees in the centre of each plot were artificially infested with a single apterous virginoparae of *D. plantaginea* using clipcages as described above. Experimental trees were inspected for naturally occurring *D. plantaginea* colonies (at an early stage of development at the time) that were removed if found to avoid interference with the established colonies. After seven days, the cages were removed. Five of these colonies per plot were covered by a mesh bag (30 × 70 cm, mesh of 300 μm, Megaview Science, Taichung, Taiwan) to exclude predators as a control treatment. At the same time, in two of the plots, 57 bean plants infested with *A. fabae* were planted underneath the trees with *D. plantaginea* colonies in a spatial arrangement in which each *D. plantaginea* colony was surrounded by three bean plants (Fig. 2). Beans were kept in the net-house and prepared following the same protocol as described previously. The number of aphids and ants associated to the established aphid colonies was recorded weekly per colony during a four-week period.

Because not all *A. fabae* survived the first week of exposure in the orchard, the uncolonized beans were used to evaluate whether ants preferred infested over uninfested bean plant. Preference was analysed using a GLM with a binomial distribution and a log link with Ants presence as response variable and *A. fabae* presence and Week as fixed factor. *D. plantaginea* and *A. fabae* colonies that did not establish in the first week were excluded from subsequent analyses. The number of ants around *D. plantaginea* colonies was analysed with a negative binomial GLM (after detecting overdispersion with a Poisson distribution) using Ant presence as response variable and Treatment and Week as fixed factors. *D. plantaginea* colony survival over time was analysed with a Cox proportional hazards regression model. Because the model tested the risk of colony predation over time, treatment was included as fixed factor and the event of colony suppression as response variable. The significance of the treatment was checked with a Wald test. The Cox model was carried out using the R package “survival”. The number of *D. plantaginea* aphids per colony was analysed using a GLMM with a negative binomial distribution (due to overdispersion) and a log link. Treatment, Week, and their first level interaction were used as fixed factors and Plot as random effect. The colony ID was nested in Plot to account for repeated aphid counts on the same colony. Pairwise comparisons of treatments in each date were performed using Tukey’s test with R package “emmeans”. Models were validated graphically as described previously.

### Chemical analyses

In June 2017, 10 apple trees and bean plants were artificially infested with *D. plantaginea* and *A. fabae* following the methodology described in the choice experiment in semi-field condition. Mesh bags were placed over the branches bearing *D. plantaginea* colonies to exclude natural enemies and ants. Aphid honeydew (1–5 μl; N = 6) was collected with microcapillary tubes from *A. fabae* and *D. plantaginea* active colonies by folding aluminium foil around the leaf hosting the colony for 6 hr. Collections were stored at −18 °C until analysis. The sugar analysis was performed via Ion Chromatography - Pulsed Amperometric Detection - Charged Aerosol Detection (IC-PAD-CAD), while the amino acid content was analysed via high precision liquid chromatography with fluorescence and diode-array detectors (HPLC-FLD-DAD). The detailed analytical protocol is presented in Appendix A. The honeydew composition of *A. fabae* and *D. plantaginea* was compared using a Willcoxon test. Because the amount of collected honeydew varied between samples, sugar and amino acids contents were normalised within each sample before the test. Compounds occurring only once were removed from the analysis.

### Laboratory and field choice test with honeydew mimic

A total of 19 *L. niger* colonies with a queen, 25–50 workers, and brood were tested in a four-choice assay with *A. fabae* and *D. plantaginea* honeydew mimics, sucrose, and water to measure ant preference. Ant colonies, collected in Erfurt (Germany) the 22^nd^ of June 2019, were purchased from Antdealer Antshop (Erfurt, Germany) and maintained in the laboratory. Ten colonies were tested twice in two consecutive weeks and 9 colonies were tested once. Colonies were kept in a climate chamber at 25 °C and 12:12 light:dark cycle and were starved for 48–50 hours before the experiment. They were kept in Petri dishes (Ø = 55 mm) with a moist charcoal-plaster bottom and a small hole in the lid to allow workers to move in and out from the nest. Each dish with a single colony was placed in a plastic box (15 × 15 × 15 cm) which served as the foraging arena. All tested stimuli were formulated at a concentration of 4% sugars and 13 nmol/ml amino acids and prepared shortly before the experiments. Twohundred µl of each solution were presented to the ants in an Eppendorf lid (Ø = 10 mm) randomly placed in a Petri dish (Ø = 55 mm) with a distance of 15 mm between solutions. The composition of honeydew mimics is reported in Table 1. The number of ants feeding from each solution was counted every ten minutes for 250 min. In addition, a cafeteria-setting experiment was conducted in the field. Seventeen ant nests were located by visual inspection in two apple orchards (and the area surrounding the orchards) situated in the Swedish University of Agricultural Sciences at Alnarp campus (Alnarp, Sweden). Nests were presented with 200 µl of each of the sugar solutions as previously described. Ants feeding from each solution were counted every five minutes for 300 min in orchard 1 (10 nests) and for 120 min in orchard 2 (7 nests). Ant count data from both experiments was pooled over time for analyses and submitted to two GLMs. The models used a negative binomial error distribution (due to overdispersion) and a log-link and included total cumulative ant sum as response variable and Treatment as factor. Multiple comparisons between treatments were conducted with Tukey’s test and model validation was carried out visually as described previously.

**Table 1:** Composition of honeydew mimics.

	A. fabae	D. plantaginea
**Sugars (mg/g)**		
Arabinose	4.65	6.12
Fructose	36.23	24.34
Glucose	42.85	35.99
Sucrose	5.48	2.72
Maltose	8.34	3.94
Melezitose	0.01	1.48
Raffinose	0.00	2.77
Erlose	1.54	10.25
Sorbitol	0.90	12.37
**Aminoacids (mg/g)**		
Alanine	7.81	1.91
Asparagine	18.18	0.00
Aspartate	21.77	6.91
Glutamate	19.37	13.10
Histidine	15.45	22.90
Isoleucine	17.41	55.18

## Results

### Choice experiment in semi-field condition

**Figure 3:**
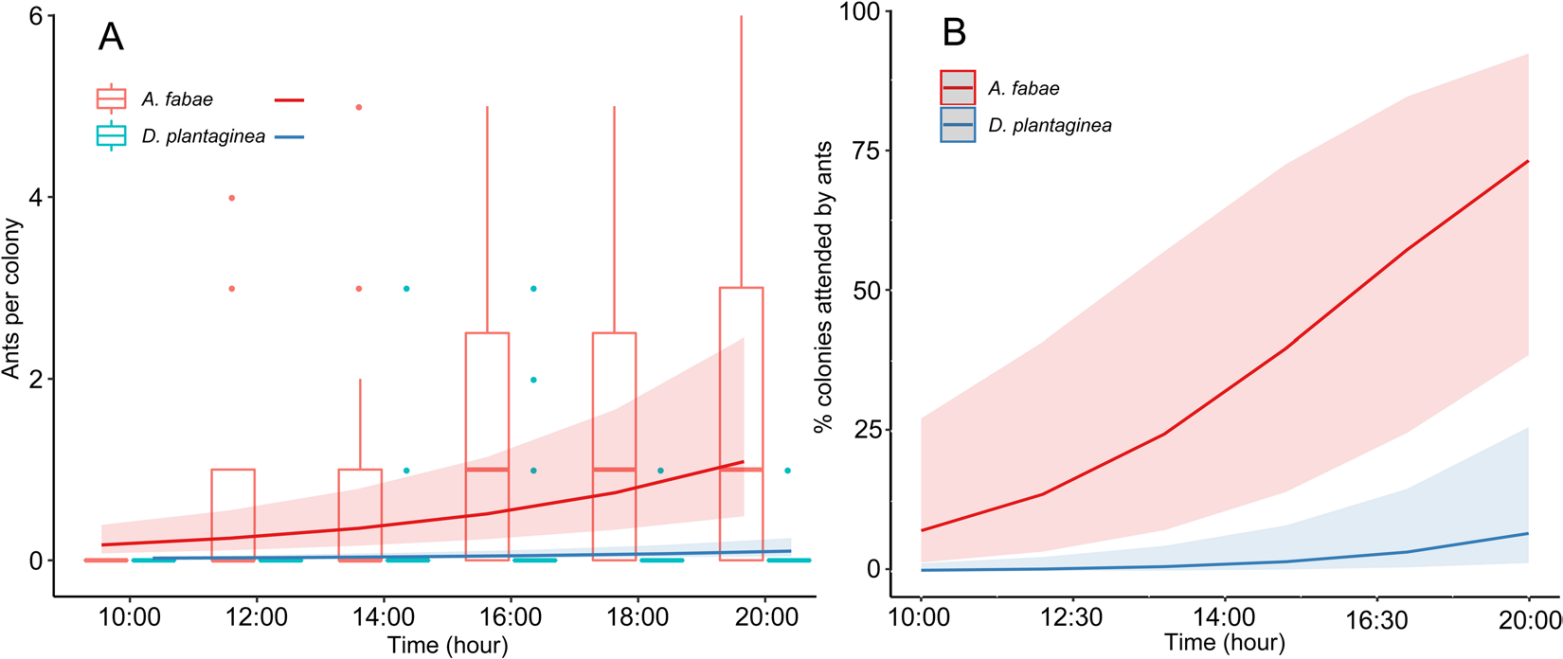
(**A**) Boxplot of the number of ants attending *D. plantaginea* and *A. fabae* colonies over time in greenhouse experiment and predicted values (±95% confidence intervals) of the GLMM. (**B**) Predicted values (±95% confidence intervals) of the GLMM representing the % of *D. plantaginea* and *A. fabae* colonies attended by *L. niger* over time.

In the greenhouse, the number of ants attending *A. fabae* was higher than for *D. plantaginea* from 10:00 until 20:00, where 1.84 ± 0.39 (mean ± SE) ants were recorded attending *A. fabae* against 0.06 ± 0.05 associated to *D. plantaginea* (Fig. 3A and Table A1). Both aphid colonies recorded an increase in ant attendance over time (Fig. 3A and Table A1). A rather stable number of ants were attending *D. plantaginea* while a considerably more pronounced increase was recorded for *A. fabae* (Fig. 3A). A similar trend was observed for the proportion of attended colonies (Fig. 3B), which increased over time with differences between species (Table A1). At the final check of the experiment, 60.0% of the *A. fabae* colonies and 6.6% of the *D. plantaginea* colonies were ant-attended.

### Field experiment

**Figure 4:**
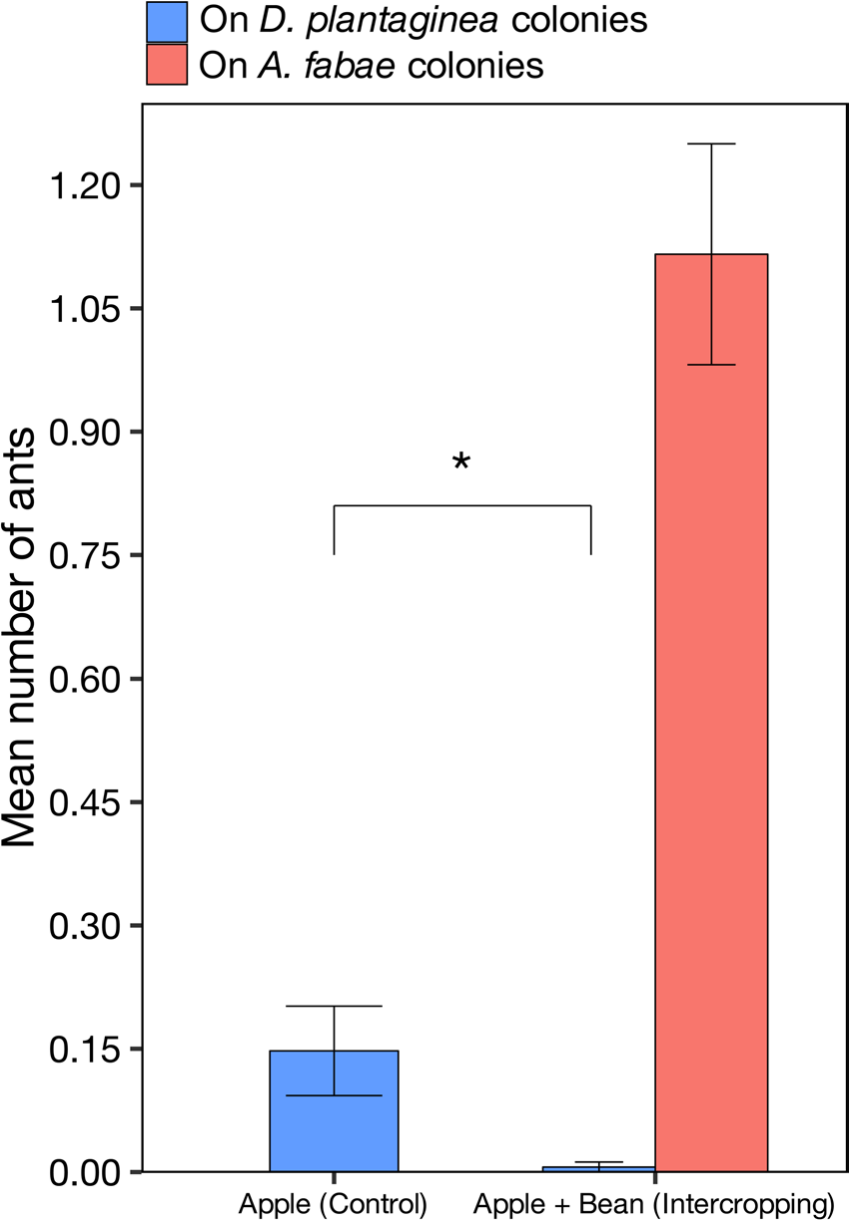
Mean number of attending ants (±SE) per plant. Asterisk indicates statistically significant differences (GLM, χ^2^ = 9.3, df = 1, *P* = 0.002).

All observed ants were identified as *L. niger* and were almost exclusively found on the infested bean plants (Fig. 4). Out of the total 114 beans planted, *A. fabae* established successfully in 73 (64.0%). Ants visited a significantly higher proportion of infested bean plants (41.0 vs 14.6%, GLM, χ^2^ = 112.8, *P* < 0.001, Table A1).

Forty-one and 39 *D. plantaginea* colonies out of 54 established adequately in the apple + bean and apple treatment, respectively. In plots without beans, 0.13 ± 0.05 (mean ± SE) ants per tree attended the *D. plantaginea* colonies. In the intercropping plots, very few ants attended *D. plantaginea* (0.01 ± 0.01) while a significantly higher number (Table A1) attended *A. fabae* (1.05 ± 0.13).

**Figure 5:**
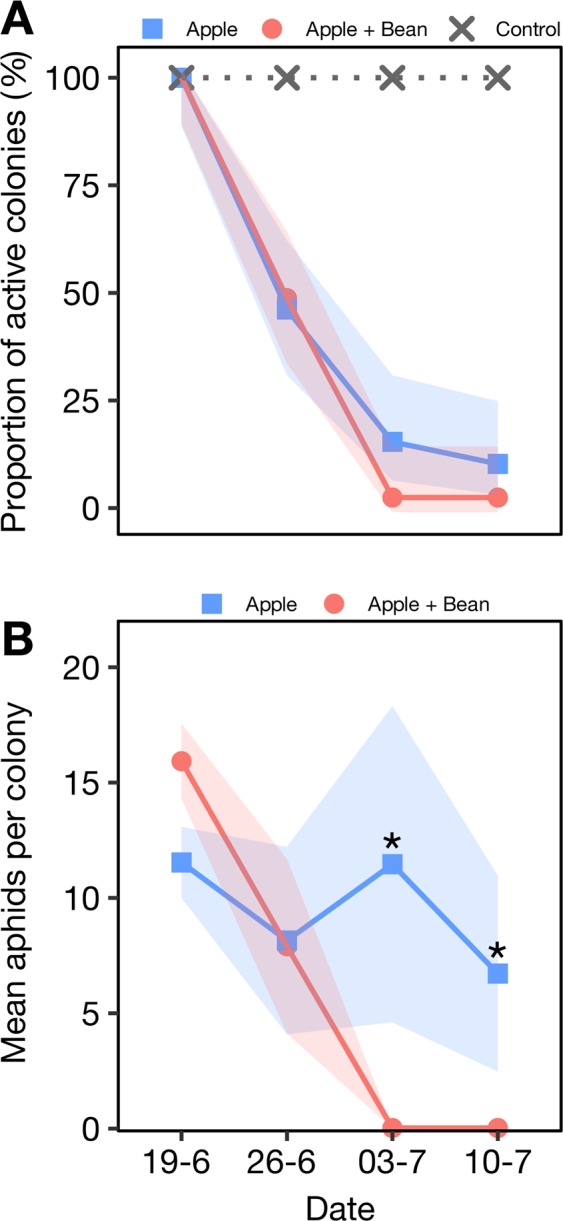
(**A**) Proportion of surviving *D. plantaginea* colonies (±95% Wald confidence interval). (**B**) Mean number of *D. plantaginea* aphids (±SE) per colony. Asterisk indicates statistically significant differences (GLMM, Tukey’s test, *P* < 0.050).

Most *D. plantaginea* colonies were predated during the first week of exposure. Only *D. plantaginea* colonies in the apple alone treatment survived to the end of the experiment, while no colonies in the apple + bean treatment survived past the third week (Fig. 5A). The number of *D. plantaginea* individuals in the established colonies decreased over time with no difference in aphid survival between treatments (Table A1, Fig. 5A,B). However, the number of aphids per colony initially established was lower in the apple + bean treatment as compared to apple alone in the last two dates of the experiment (Fig. 5B).

### Chemical analysis of aphid honeydew

**Figure 6:**
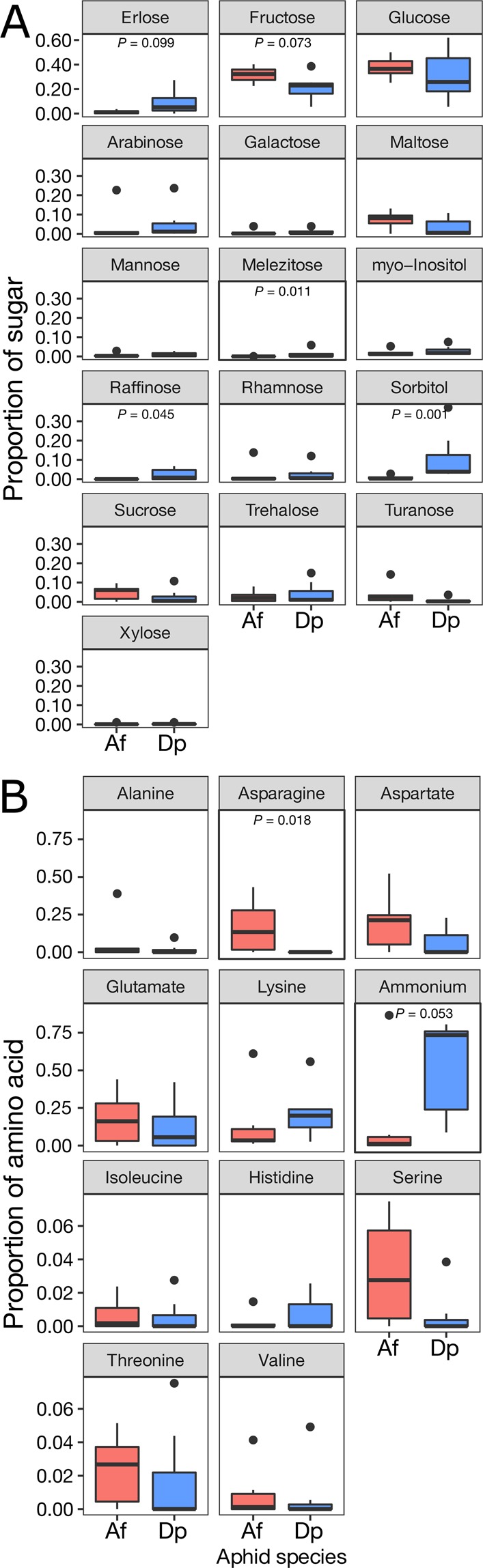
Boxplot of normalized (**A**) sugar content and (**B**) amino acids content (as proportion) in honeydew collected from *A. fabae* (Af) and *D. plantaginea* (Dp). Only *P*-values < 0.100 are shown (Wilcoxon test). The amino acids arginine, citrulline, glutamine, leucine, phenylalanine and tyrosine were not included in the analysis because they were detected only once.

The analysis of the honeydew disclosed differences between species (Fig. 6). The monosaccharide glucose was the dominating sugar of both species, followed by the monosaccharide fructose (Fig. 6A). Among trisaccharides, raffinose was detected exclusively in *D. plantaginea*’s honeydew, and the honeydew-specific melezitose, although found in both species, showed a higher content in the *D. plantaginea* honeydew. Other sugars such as those coming from hemicellulose breakdown or conjugated in other molecules, i.e. the monosaccharides arabinose, galactose, mannose, rhamnose, xylose and the disaccharides turanose and trehalose were minor components of the honeydew of both species. The sugar alcohol sorbitol was found in significantly higher proportion in *D. plantaginea* (Fig. 6A). Myo-inositol was the only carbocyclic sugar detected in the honeydew of both aphids.

Glutamate was the most common amino acid found in both species (Fig. 6B). Asparagine showed a higher content in the *A. fabae* in comparison to the *D. plantaginea* honeydew. The normalized content of ammonium was instead higher in the honeydew from *D. plantaginea*, although not significantly different (Table A1). The content of serine, aspartate, glutamate, threonine and valine tended to be higher but not significantly different in the bean aphid, while the opposite was measured for isoleucine (Fig. 6B). Lysine and histidine tended to be higher but not different for the rosy apple aphid. Alanine showed a low content in both species.

### Laboratory and field choice test with honeydew mimic

**Figure 7:**
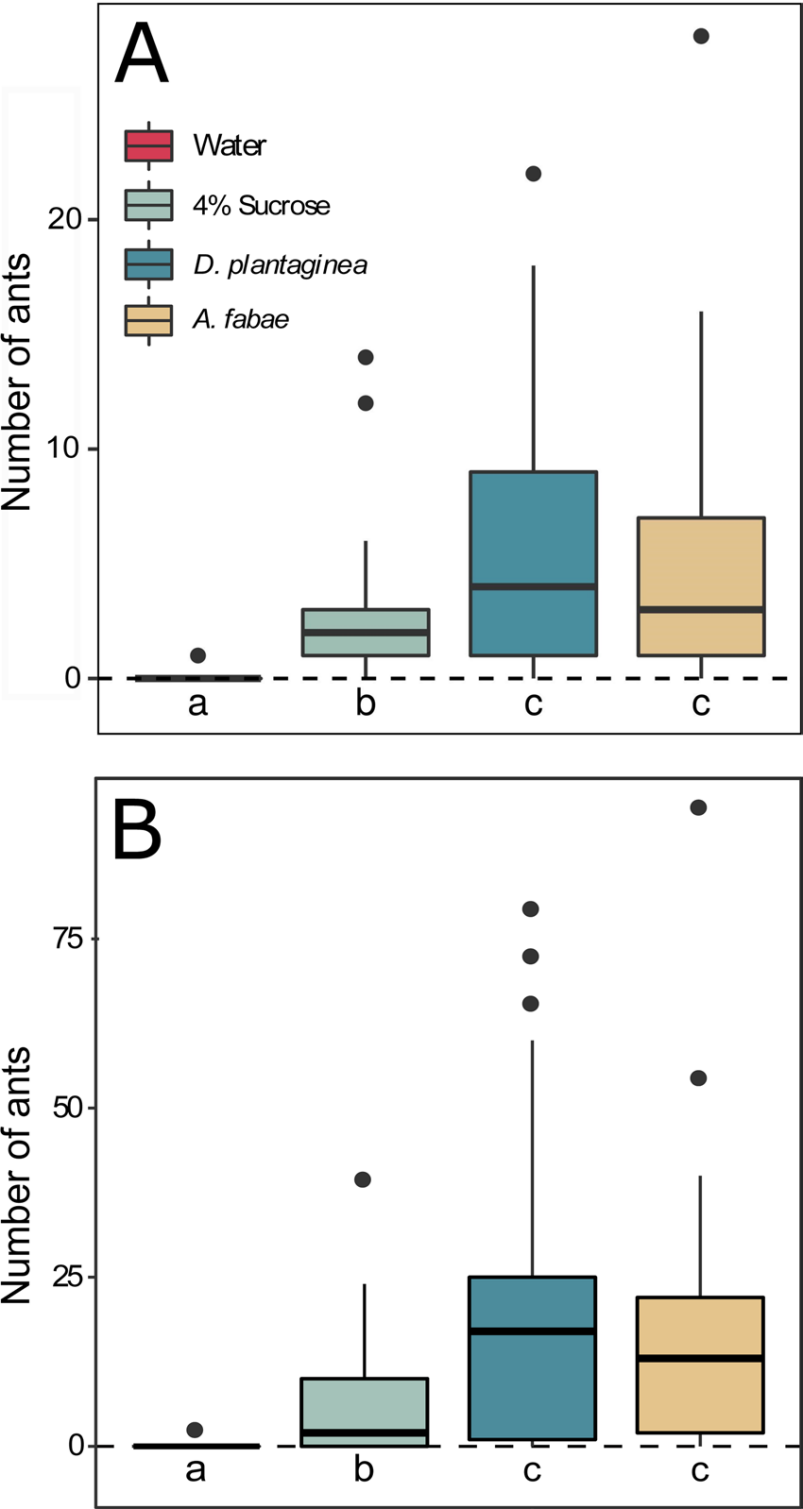
Boxplot of *L. niger* visiting water, sucrose solution and honeydew mimic of *A. fabae* and *D. plantaginea* in the (**A**) laboratory and (**B**) field experiments. Different letters indicate significant differences (GLM, *P* < 0.050).

*L. niger* did not discriminate between honeydew mimics of *A. fabae* and *D. plantaginea* in both the laboratory and field choice assays (Fig. 7). Sucrose was less preferred than any mimic, while water attracted only a few ants with very similar results in the laboratory and the field (Fig. 7).

## Discussion

The possibility of exploiting differential ant attendance to generate new intercropping or selected plants combinations represents a practical opportunity to increase the resilience of cultivated systems. In this study we confirmed the initial prediction, i.e. that the supplement of a specific plant within a perennial cropping system would divert ant attendance from an aphid attacking the perennial crop to an aphid feeding on the newly introduced plant. The effect of intercropping on ant was initially measured in the greenhouse and subsequently confirmed in a full field setting. In a previous study, intercropping was associated with season-long reductions in ant activity in a pecan tree orchard^21^, although influence on a specific pest was not shown.

Previous studies showed a relationship between reduction of ant attendance, increase of antagonists in colonies, and drop in aphid populations in apple and other cropping systems. Nagy *et al*.^38^ used different artificial feeders and sugars attaining a reduction of *D. plantaginea* by reducing *L. niger* attendance in all cases. They reported the highest ant diversion and aphid reduction for sucrose feeders attached to the tree trunk and argued that proximity to the ant nest, as opposed to feeders on the branches, may increase diversion. A similar effect was observed for the mutualism between *Aphis spiraecola* Patch and *Lasius grandis* Forel^39^. A solution comprising different sugars decreased attendance and aphid populations. However, an increase in antagonists was only noticeable when feeders were placed on branches. Our results, using a sentinel colony methodology, did not evidence a significantly higher biological control in terms of colony reduction over time. Nonetheless, colonies remained active in the control treatment the last two weeks of assessment whilst they were almost eradicated in the presence of bean plants (Fig. 5). The same active colonies grew larger over time under ant-attendance. A pronounced drop in colonies during the first two weeks in both treatments can probably explain the lack of differences observed. A high predator population, in particular of *Anthocoris nemorum* (L.), that peaks at this time of the year, could have outcompeted *L. niger* in the location of the small aphid colonies after clipcage removal. This could have partially prevented the establishment of mutualism in the control treatment in our field setting. Porcel *et al*.^18^ showed the efficiency of this natural enemy to quickly locate *D. plantaginea* colonies that are not under ant-attendance protection. Additionally, it is possible that ants had already established trails to track naturally occurring colonies at the time of year the experiment was carried out. Such trails could have deterred the presence of antagonists in the apple + bean treatment diluting the treatment effect. Given the complexity at the field level, further research should evaluate the population of natural enemies, the effect of temperature and the possible influence of already established non-inoculated aphids on aphid-ant-natural enemies interaction.

Fischer *et al*.^22^ showed that there is a competition for the mutualistic services of *L. niger* between the aphid *A. fabae, Metopeurum fuscoviride* Stroyan and *Brachycaudus cardui* (L.) utilizing the same plant *Tanacetum vulgare*. While *M. fuscoviride* was the most attended species, *A. fabae* was the least intensively attended. Authors argued that qualitative and quantitative differences in honeydew determine the aphid’s selective attractiveness to ants, with the least competitive species being predated by *L. niger*. Although in our experiments with sentinel colonies we did not observe predation by ants neither upon *A. fabae* nor upon *D. plantaginea* (data not shown), the spatial isolation of the species may have played a role in moderating such competition. Probably, *L. niger* simply visited more intensively *A. fabae* to optimize the rate of honeydew harvest, made possible by a shorter distance from the nest compared to *D. plantaginea*^23^. As an alternative explanation, *L. niger* may prefer bean over apple due to the presence of extra floral nectaries.

Offenberg^24^ observed an increased predation on *A. fabae* when artificial nectaries were offered to *L. niger*, whereas alternative prey had no significant effect. Consequently, a higher density of *V. faba* in the orchard or the addition of artificial nectaries may restore the balance. The shift of ants from extrafloral nectaries to aphid colonies seems to occur when the total reward from aphids will exceed that from extrafloral nectaries^25^.

The chemical analysis of the honeydew highlighted a rather specific profile for the two aphid species, with a higher content of the trisaccharides melezitose and raffinose but a lower presence of 1 out of 17 amino acids (asparagine) in *D. plantaginea*’s honeydew. This result is in contrast with Woodring *et al*.^26^, who found that aphid species with the highest total amino acid concentration in the honeydew always had the highest concentration of sugars. It seems however, that the content of specific saccharides plays a major role in ant attraction. Woodring *et al*.^27^ discussed that the sugar composition of the honeydew of the ant-attended *M. fuscoviride* indicated a rapid digestion of sucrose into glucose and fructose, and the simultaneous synthesis of considerable amounts of melezitose and some trehalose. Melezitose, a trisaccharide synthetized by aphids, has been reported to be used by ant scouts as a cue indicative of a long-lasting productive resource for collective exploitation and defence against competitors or aphid predators^28^. Trail mark laying was exclusively triggered by raffinose, sucrose and melezitose. A plant-specific honeydew production seems to be common in polyphagous species such as *A. fabae*, where the quality of the plant directly affects honeydew composition and indirectly ant attendance^30^. In our experiment, an increased preference for *A. fabae* could perhaps have been achieved by selecting plants with a higher release of melezitose, because *A. fabae* from *E. europaeus* produces a honeydew low in this compound^29,30^. However, *A. fabae* was more tended than *D. plantaginea* in both greenhouse and field experiments in spite of releasing less melezitose than *D. plantaginea*. The role of melezitose in the establishment of the mutualism is not entirely clear for *A. fabae*, because low-producing clones manage to attain high frequency of attendance^31^. Perhaps also the quality and the density of extrafloral nectaries on *V. faba* as well as the size of the colony and the related amount of produced honeydew need to be taken into account when evaluating the efficacy of ant diversion in apple-bean intercropping^32^.

It is nonetheless intriguing that in both our greenhouse and field experiments, *A. fabae* attracted a large majority of *L. niger*, disregarding the lower content of raffinose and sucrose and the complete absence of melezitose. The higher level of the sugar alcohol sorbitol in *D. plantaginea*’s honeydew is somehow expected, as it is often found in the apple phloem. The carbocyclic sugar myo-inositol has not previously been retrieved from aphid honeydew and it seems to be related to glucose metabolism and cold tolerance in hemipterans^33,34^.

The amino acid composition is reported as a relevant factor in the establishment of ant-aphid mutualism^35^. Whilst amino acid analyses of *A. fabae* honeydew are available in the literature, no information could be found on *D. plantaginea*’s honeydew profile. In our study we retrieved 6 major, 5 minor and 6 “in traces” amino acids. Two of the major ones, asparagine and isoleucine, differed substantially between the aphid species. Schillewaert *et al*.^29^ reported arginine, asparagine and glycine as major amino acids in *A. fabae*, whereas isoleucine and 13 other amino acids were detected at an intermediate or lower content. Although the lack of information on the possible effect of specific amino acids such as asparagine and isoleucine on ant preference does not allow us to interpret further our result, it appears that ant preference falls under the category of context-dependent behaviours. Because *L. niger* did not discriminate between *A. fabae* and *D. plantaginea* honeydew mimics in our laboratory and field assays, we argue that the result observed in the intercropping experiment is mainly driven by other factors than honeydew contents. It is possible that a combination of factors such as honeydew profile, aphid colony growth and distance of the colony from the ant nest may affect the final outcome of the mutualism. In addition, plant architecture may shape the distance between colonies, which could in turn affect the foraging activity of ants^36^. Because of plant quality in deciduous trees may change with phenological development, the ant-aphid interaction observed in our study could be differentially shaped at a later stage in the season.

Recent studies showed that *L. niger* scouts were attracted not only to the complete plant system and honeydew, but also to the microorganisms in the absence of plant or honeydew; more specifically to a bacterium from *A. fabae*’s honeydew. *Staphylococcus xylosus* emits a blend of volatiles that attract ant scouts^37^. In the present study we did not examine the possible effect of honeydew volatiles on ant attendance. However, it might be relevant to measure such attraction in order to shed additional light on the factors regulating the increased preference of *L. niger* towards *A. fabae*.

An aspect to consider is that the intensity of attendance reduction observed in Nagy *et al*.^38^ and Wackers *et al*.^39^, by using artificial sucrose feeders, is comparable to what we report in this study (Fig. 4). This leads us to infer that, under certain circumstances, equivalent biological control increases may be obtained with the natural system that we present here.

In this study we demonstrated the potential of intercropping an aphid*-*bearing plant in orchards as a method to reduce ant attendance in aphid colonies in order to increase biological control. Future studies should clarify whether the same effect can be achieved by only sowing beans without inoculating the aphid. Additionally, alternative companion plants and aphids would be needed to increase diversity and provide a more robust system as aphid population can vary between years. We conclude that the concept of using a plant capable of diverting ant attendance from the main cultivated crops holds the potential to be included in conservation biological control of horticultural crops as a bottom-up approach to enhance the action of natural enemies. Together with other ecosystem-based measures, intercropping should be thus evaluated when planning ecosystem redesign to decrease the use of synthetic inputs such as pesticides.

## Supplementary information

Supplementary Information.

Supplementary Information2.
