# Supplementary material for: Aphid-infested beans divert ant attendance from the rosy apple aphid in apple-bean intercropping
Source: Sci Rep. 2020 May 19;10:8209. doi: 10.1038/s41598-020-64973-7 (PMC7237440; doi:10.1038/s41598-020-64973-7)
Supplement: Supplementary file 1 — Supplementary Information. [file 41598_2020_64973_MOESM1_ESM.docx]

**Aphid-infested beans divert ant attendance from the rosy apple aphid in** **apple-bean intercropping**

Joakim Pålsson, Mario Porcel, Mette Frimodt Hansen, Joachim Offenberg, Tiziana Nardin, Roberto Larcher & Marco Tasin

Table A.1. Statistical analyses results.

| Model | Model type | Factors | Χ^2^/W | Df | *p*-value |
| --- | --- | --- | --- | --- | --- |
| Number of *L. niger*  on colonies (greenhouse) | Poisson  GLMM | Aphid | 94.6 | 1 | < 0.001 |
|  |  | Time | 71.3 | 1 | < 0.001 |
| Proportion of colonies with *L. niger* (greenhouse) | Binomial GLMM | Aphid | 47.2 | 1 | < 0.001 |
|  |  | Time | 31.5 | 1 | < 0.001 |
| *L. niger*  preference for un/infested beans (field) | Binomial GLM | *A. fabae* presence | 112.8 | 1 | < 0.001 |
|  |  | Week | 0.1 | 1 | 0.745 |
| Ants around  *D. plantaginea* (field) | Negative binomial GLM | Treatment | 9.3 | 1 | 0.002 |
|  |  | Week | 0.0 | 1 | 0.979 |
| Number *D. plantaginea* per colony | Negative binomial GLMM | Treatment | 0.0 | 1 | 0.886 |
|  |  | Week | 163.8 | 3 | <0.001 |
|  |  | Treatment:Week | 17.8 | 3 | <0.001 |
| *D. plantaginea* Survival | Cox Model | Aphids death | 1.4 | 1 | 0.236 |
| Sugar in Honeydew | Wilcoxon | Arabinose | 10 |  | 0.131 |
|  |  | Erlose | 9 |  | 0.099 |
|  |  | Fructose | 34 |  | 0.073 |
|  |  | Galactose | 10 |  | 0.138 |
|  |  | Glucose | 26 |  | 0.534 |
|  |  | Maltose | 28.5 |  | 0.311 |
|  |  | Mannose | 22 |  | 0.945 |
|  |  | Melezitose | 3.5 |  | 0.011 |
|  |  | myo-Inositol | 13 |  | 0.295 |
|  |  | Raffinose | 9 |  | 0.045 |
|  |  | Rhamnose | 13 |  | 0.281 |
|  |  | Sorbitol | 0 |  | 0.001 |
|  |  | Sucrose | 27 |  | 0.419 |
|  |  | Trehalose | 21 |  | 1.000 |
|  |  | Turanose | 31 |  | 0.172 |
|  |  | Xylose | 17 |  | 0.628 |
| Amino acid in honeydew | Wilcoxon | Alanine | 24.5 |  | 0.625 |
|  |  | Arginine | 21 |  | 1.000 |
|  |  | Asparagine | 35 |  | 0.018 |
|  |  | Aspartate | 29 |  | 0.244 |
|  |  | Citrulline | 24.5 |  | 0.355 |
|  |  | Glutamine | 18 |  | 0.440 |
|  |  | Glutamate | 25 |  | 0.607 |
|  |  | Histicine | 17 |  | 0.569 |
|  |  | Isoleucine | 24.5 |  | 0.625 |
|  |  | Leucine | 24.5 |  | 0.355 |
|  |  | Lysine | 12 |  | 0.234 |
|  |  | Ammonium | 7 |  | 0.053 |
|  |  | Phenylalanine | 24.5 |  | 0.355 |
|  |  | Serine | 31 |  | 0.140 |
|  |  | Threonine | 26 |  | 0.485 |
|  |  | Tyrosine | 22 |  | 0.910 |
|  |  | Valine | 24.5 |  | 0.625 |
