# Supplementary material for: Aphid-infested beans divert ant attendance from the rosy apple aphid in apple-bean intercropping
Source: Sci Rep. 2020 May 19;10:8209. doi: 10.1038/s41598-020-64973-7 (PMC7237440; doi:10.1038/s41598-020-64973-7)
Supplement: Supplementary file 2 — Supplementary Information2. [file 41598_2020_64973_MOESM2_ESM.docx]

**Aphid-infested beans divert ant attendance from the rosy apple aphid in** **apple-bean intercropping**

Joakim Pålsson, Mario Porcel, Mette Frimodt Hansen, Joachim Offenberg, Tiziana Nardin, Roberto Larcher & Marco Tasin

**Chemical analyses of the honeydew**

**Ion Chromatography - Pulsed Amperometric Detection - Charged Aerosol Detection (IC-PAD-CAD) sugar analysis:**

The standards used for quantitative determination D-(-)-arabinose (98%), erlose (≥94%), D-(+)-fructose (99%), D-(+)-galactose (97%), D-(+)-glucose (99.5%), maltitol (≥98%), maltose monohydrate (98%), L-(-)-mannose (>99%), D-(+)-melezitose hydrate (99%), myo-inositol (99%), D-(+)-raffinose, L-(-)-rhamnose monohydrate (99%), D-sorbitol (98%), sucrose (≥99.5%), D-(+)-trehalose dihydrate, D-(+)-turanose (≥98%) and D-(+)-xylose (99%) were purchased from Sigma-Aldrich (Saint Louis, Missouri, Stati Uniti). A stock solution of sugars was prepared by weighing 10 mg of each one standard powder into 100 ml of water (100 mg/L), then, mix solutions were obtained for calibration by diluting the stock solution at 0.2, 0.5, 2, 4, 25, 100, 250 mg/L and injecting 5 µL of each one. The aphid honeydew samples were prepared by collecting and weighing the content of the microcapillary in an HPLC-vial and adding 0.5 mL of water and methanol (90:10, v/v). Sample, standard and eluent were prepared with ultrapure water >18 Mohm/cm obtained using an AriumVR Pro Lab Water System (Sartorius AG, Goettingen, Germany).

Chromatographic separation was performed using an ICS 5000 ion chromatography system (Dionex; Thermo Scientific, Waltham, USA) equipped with an eluent generator, a pulse amperometric detector (PAD) consisting of a gold working electrode and a palladium counter electrode, and a charged aerosol detector (CAD) according to Di Lella et al. (2019). The separation of carbohydrates was carried out on a CarboPac PA200 (3 x 250 mm) analytical column, which was preceded by a CarboPac PA200 (3 x 50 mm) guard column (Dionex). Both columns were operated at a constant temperature of 30 °C. Chromatographic elution was carried out at a flow rate of 0.4 mL/min using an eluent generator that allowed the automatic production of potassium hydroxide (KOH) eluent, controlling the electrical current applied to the electrolysis of deionized water. Isocratic KOH elution at 0.1 mM was run from 0 to 15.5 min, then gradient elution was performed from 0.1 to 100 mM, from 15.5 to 21.5 min and held until 27.5 min. KOH concentration was then reduced to 0.1 mM, enabling column equilibration for 5 min. Deionized water was constantly purged with helium to avoid the formation of carbonate. Carbohydrate detection was achieved using PAD with the working pulse potential quaternary curve with reference to a saturated Ag/AgCl reference electrode. For CAD monitoring, a low filter was used and nitrogen gas pressure was adjusted to 35 psi. The eluent, rich in KOH, was not compatible with the CAD detector, and to overcome this problem an anionic electrochemical regenerator suppressor (AERS 500; 2 mm) was used to remove nonvolatile solutes. The detection limit for sugars was estimated at 1 mg/kg each one (3 times the standard deviation of the standard content measured repeating 10 times the analysis of a sample at unquantifiable content and multiplied by the sample dilutions).

**High precision liquid chromatography with fluorescence and diode-array detectors (HPLC-FLD-DAD) analysis of amino acids:**

HPLC-grade methanol and orto-phthaldialdeyde (>99%; OPA) were purchased from Fluka (St. Louis, MO, USA). Hydrochloric acid (37%, HCl), 2-mercaptoethanol (99%), sodium acetate anhydrous (>99%), sodium tetraborate decahydrate (>99.5%), the standard used for the quantification L-alanine (>98%; ala), γ-aminobutyric acid (> 99%; GABA), ammonium nitrate (>98%; NH4), L-arginine (>98%; arg), L-asparagine (>98%; asn), L-aspartic acid (>98%; asp), ethanolamine (>99.5%), β-glutamic acid (>98%; internal standard), L-glutamic acid (>99%; glu), glycine (>99%; gly), L-histidine (>98%; his), L-isoleucine (>98%; ile), L-leucine (>98%; leu), L-lysine (>98%; lys), L-methionine (>98%; met), L-ornithine monohydrochloride (>99%; orn), L-phenylalanine (>98%; phe), L-serine (>99%; ser), L-tryptophan (>98%; trp), L-tyrosine (>98%; tyr), L-valine (>98%; val) and L-threonine (>98%; thr) were purchased from Sigma-Aldrich (Saint Louis, Missouri, Stati Uniti). A stock solution of amino acid was prepared by weighing 10 mg of each standard powder into 100 ml of HCl 0.1N (100 mg/L), then calibration solutions were obtained by diluting the stock solution at 0.05, 1, 20, 80 and 100 mg/L. 99 µL of sample prepared for sugar analysis were added with 1 µL of internal standard (β-glutamic acid, 50 mg/L final concentration) and put in HPLC-vial. Sample, standard and eluent were prepared with ultrapure water >18 Mohm/cm obtained using an AriumVR Pro Lab Water System (Sartorius AG, Goettingen, Germany).

The measures were performed using an UHPLC Ultimate 3000 (Thermo Scientific, Waltham, Massachusetts, Stati Uniti) equipped with a fluorescence detector (Ex = 336 nm, Em = 445 nm). Separation was carried out with sodium acetate 0.05 M (adjusted pH 7.5; eluent A) and methanol (eluent B) using a Chromolith Performance RP-18e column (100 x 4.6 mm; Merk, Darmstadt, Germania) with Chromolith RP-18e guard cartridge (10 x 4.6 mm; Merk) thermostated at 40 °C. The flow rate was set at 2 ml/min. The analytical gradient for eluent B was: 1 min at 0%, to 20% in 10 min, to 50% in 6 min, to 100% in 4.5 min, held at 100% for 4.5 min for cleaning, and to 0% for reconditioning in 2 min. The sample (10 μl), kept at 10°C by the autosampler, was automatically introduced in the loop, added of 10 μl derivatizing solution (4.5 g/l of OPA in sodium tetraborate 0.1 M pH 10.5, 10% of methanol and 2% of 2-mercaptoethanol), mixed for 30 seconds, and injected. The detection limit for amino acid was estimated at 5 mg/kg each one (3 times the standard deviation of the standard content measured repeating 10 times the analysis of a sample at unquantifiable content and multiplied by the sample dilutions).
